# The adipokine FABP4 is a key regulator of neonatal glucose homeostasis

**DOI:** 10.1172/jci.insight.138288

**Published:** 2021-10-22

**Authors:** Idit Ron, Reut Kassif Lerner, Moran Rathaus, Rinat Livne, Sophie Ron, Ehud Barhod, Rina Hemi, Amit Tirosh, Tzipora Strauss, Keren Ofir, Ido Goldstein, Itai M. Pessach, Amir Tirosh

**Affiliations:** 1 The Dalia and David Arabov Endocrinology and Diabetes Research Center, Division of Endocrinology, Diabetes and Metabolism, Tel HaShomer, Israel.; 2 Department of Pediatrics, Edmond and Lily Safra Children’s Hospital, Sheba Medical Center, Tel HaShomer, Israel.; 3 Sackler School of Medicine, Tel Aviv University, Tel Aviv, Israel.; 4 Endocrinology Laboratories,; 5 Endocrine Cancer Genomics Center, Sheba Medical Center, Tel HaShomer, Israel.; 6 Department of Neonatology, Edmond and Lily Safra Children’s Hospital, Sheba Medical Center, Tel HaShomer, Israel.; 7 Department of Obstetrics and Gynecology, Sheba Medical Center, Tel HaShomer, Israel.; 8 Institute of Biochemistry, Food Science and Nutrition, The Robert H. Smith Faculty of Agriculture, Food and Environment, Hebrew University of Jerusalem, Rehovot, Israel.; 9 Department of Pediatric Intensive Care, Edmond and Lily Safra Children’s Hospital, Sheba Medical Center, Tel HaShomer, Israel.

**Keywords:** Endocrinology, Metabolism, Adipose tissue, Glucose metabolism

## Abstract

During pregnancy, fetal glucose production is suppressed, with rapid activation immediately postpartum. Fatty acid–binding protein 4 (FABP4) was recently demonstrated as a regulator of hepatic glucose production and systemic metabolism in animal models. Here, we studied the role of FABP4 in regulating neonatal glucose hemostasis. Serum samples were collected from pregnant women with normoglycemia or gestational diabetes at term, from the umbilical circulation, and from the newborns within 6 hours of life. The level of FABP4 was higher in the fetal versus maternal circulation, with a further rise in neonates after birth of approximately 3-fold. Neonatal FABP4 inversely correlated with blood glucose, with an approximately 10-fold increase of FABP4 in hypoglycemic neonates. When studied in mice, blood glucose of 12-hour-old WT, *Fabp4*^–/+^, and *Fabp4*^–/–^ littermate mice was 59 ± 13 mg/dL, 50 ± 11 mg/dL, and 43 ± 11 mg/dL, respectively. Similar to our observations in humans, FABP4 levels in WT mouse neonates were approximately 8-fold higher compared with those in adult mice. RNA sequencing of the neonatal liver suggested altered expression of multiple glucagon-regulated pathways in *Fabp4*^–/–^ mice. Indeed, *Fabp4*^–/–^ liver glycogen was inappropriately intact, despite a marked hypoglycemia, with rapid restoration of normoglycemia upon injection of recombinant FABP4. Our data suggest an important biological role for the adipokine FABP4 in the orchestrated regulation of postnatal glucose metabolism.

## Introduction

Hepatic glucose production, a key metabolic pathway in maintaining glucose homeostasis, is tightly regulated by a complex hormonal network (1–5). During normal pregnancy, glucose, the main energy source for fetal development, is continuously transferred through the placenta to ensure adequate glucose availability to the fetus. Therefore, under normal conditions, given the intensive anabolic queues in utero, the rapidly growing fetus relies entirely on maternal glucose supply, and hepatic gluconeogenesis, an energy consuming pathway, is profoundly inhibited (6). The abrupt termination of umbilical glucose supply during delivery induces an endocrine stress response characterized by a significant rise in plasma catecholamines, glucagon, and cortisol, with a concomitant decrease in insulin level (7, 8) and an increase in liver expression of the glucagon receptor (9, 10). This rapid shift in the ratio of insulin to counterregulatory hormones triggers the induction of hepatic glycogenolysis (9, 10) and simultaneous induction in gene expression of phosphoenolpyruvate carboxykinase (PEPCK), the rate-limiting enzyme in gluconeogenesis (11). To date, the exact mechanism(s) leading to the rapid induction of hepatic glucose production and release are not fully understood (12).

Fatty acid–binding protein 4 (FABP4) is one of the most abundant proteins in adipocytes, and, in addition to its intracellular biological actions (13), it also acts as an adipokine, promoting hepatic expression of key gluconeogenetic enzymes and glucose production (14). The key role of FABP4 in regulating systemic metabolism could also be evidenced by various biochemical and genetic studies demonstrating an important role for FABP4 levels in metabolic diseases (13–16). Thus, we hypothesized that FABP4 participates in neonatal glucose homeostasis by regulating the hepatic glucose production “switch on” immediately after birth. To assess this, we studied the dynamics in serum levels of FABP4 from maternal, fetal, and neonatal samples within the first few hours of life. We demonstrated that the FABP4 level is higher in fetal circulation than in maternal circulation, with a further increase by approximately 3-fold in the first few hours after delivery. In addition, neonatal FABP4 levels inversely correlated with blood glucose, with the highest levels recorded in neonates experiencing hypoglycemia. Furthermore, we studied the direct effect of FABP4 on glucose homeostasis in *Fabp4*-knockout (*Fabp4*^–/–^) neonates and demonstrated lower blood glucose in FABP4-haploinsufficient mice, with a further drop in blood glucose, with the complete absence of FABP4. Corresponding to results in humans, WT (*Fabp4^WT^*) mouse neonates showed a significant increase of about 8-fold in plasma FABP4. An unbiased screen of the neonatal liver transcriptome suggested the regulation of multiple glucose and lipid metabolic pathways with enhanced glycogen metabolism. Indeed, liver glycogen was inappropriately intact in hypoglycemic *Fabp4*^–/–^ neonates, with rapid recovery of blood glucose with injection of recombinant FABP4 (recFABP4), indicating its direct contribution in postnatal blood glucose maintenance. Thus, our results highlight the importance of FABP4 as a significant factor in regulating postnatal systemic glucose metabolism, as part of the orchestrated hormonal and metabolic adaptive response to maintain glucose homeostasis in the immediate postnatal period.

## Results

### Maternal circulating FABP4 level is increased in gestational diabetes mellitus.

**Figure 1:**
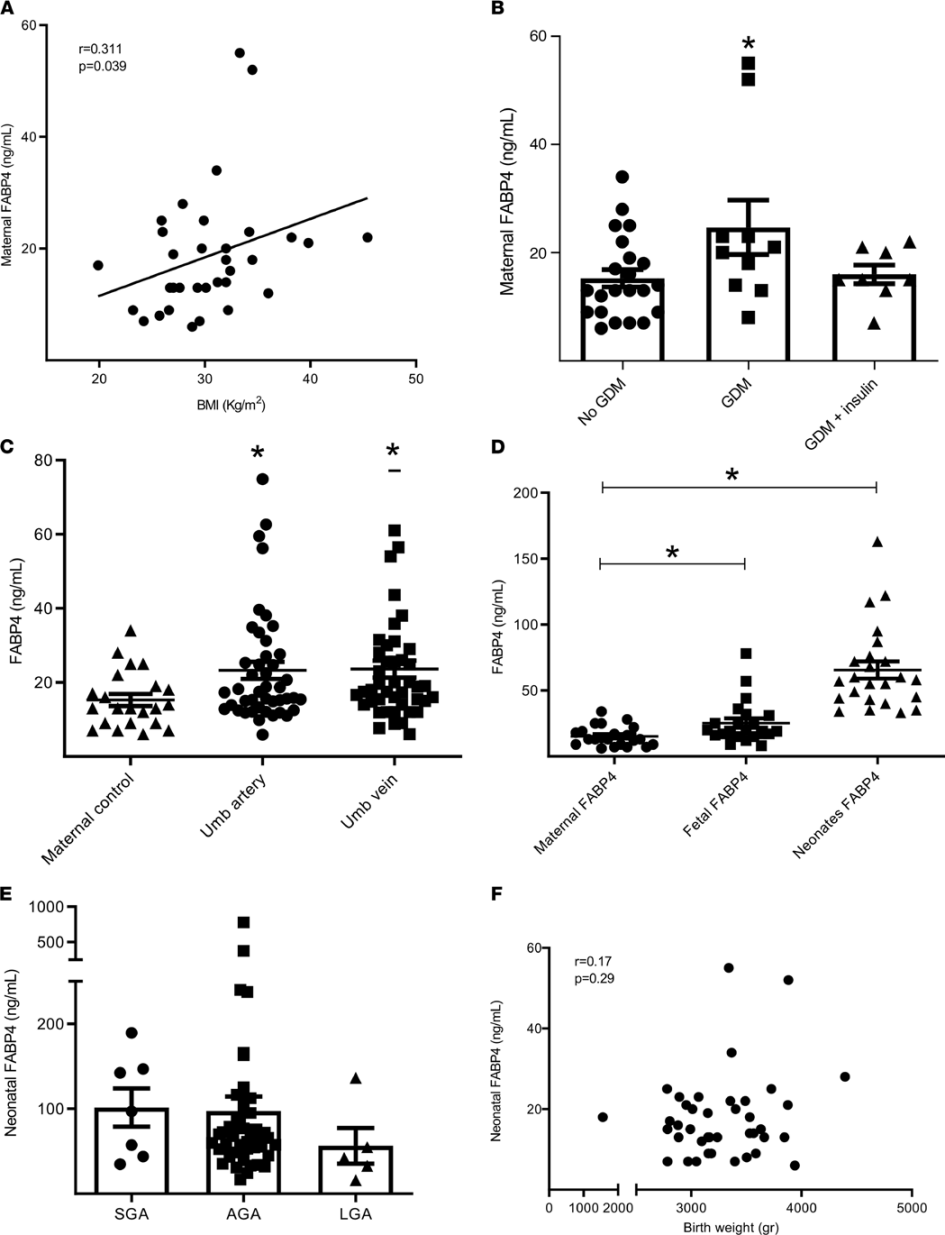
Maternal, fetal, and neonatal FABP4 circulating levels. Serum samples were collected from 22 normoglycemic pregnant women and 18 women with GDM. All samples were collected at term, immediately before delivery. (**A**) FABP4 serum concentrations were determined using an ELISA assay and were correlated to BMI. (**B**) Comparison between FABP4 levels in normoglycemic pregnant woman (*n* = 22) and women with GDMA1 (*n* = 10) or GDMA2 (*n* = 8). (**C**) Serum samples, collected from umbilical artery (*n* = 22) and vein (*n* = 22) immediately after delivery of normoglycemic women were analyzed for FABP4 levels and compared with (normoglycemic) maternal (*n* = 22) concentrations. (**D**) Serum samples collected from neonates within the first few hours of life (*n* = 24) were analyzed for FABP4 levels and compared with fetal (*n* = 22) and maternal levels (*n* = 22). (**E**) FABP4 levels stratified to neonates who were small (SGA) (*n* = 7), appropriate (AGA) (*n* = 48), and large (LGA) (*n* = 5) for gestational age. (**F**) Birth weight of 40 neonates was correlated to FABP4 serum concentrations. Statistical analysis includes Spearman’s correlation test (**A** and **F**) and 1-way ANOVA (**B–E**). Data are shown as the mean ± SEM. *P < 0.05.

To study the dynamics in FABP4 serum levels in mothers and offspring, we first tested FABP4 serum levels in normoglycemic pregnant women (control) and women with gestational diabetes mellitus (GDM). All samples were collected immediately before delivery. In accordance with previous publications (17–20), maternal FABP4 serum concentration correlated with BMI (Spearman’s *r* = 0.311, *P* = 0.039) (Figure 1A), and women with GDM had significantly higher levels of FABP4 as compared with normoglycemic pregnant women (24.7 ± 15.9 ng/mL vs. 15.2 ± 7.6 ng/mL, *P* = 0.0146). Of note, women with insulin-treated GDM (GDMA2) did not exhibit an increase in FABP4 levels; their levels were comparable to those of normoglycemic pregnant women (Figure 1B). This observation may be due, at least in part, to the potent suppressive effect of insulin on FABP4 secretion (21, 22).

### Circulating levels of FABP4 in maternal, fetal, and neonatal circulations.

We further studied FABP4 levels in fetal serum obtained from sampling of the umbilical artery and vein immediately after birth. A significantly higher FABP4 concentration was observed in both umbilical arteries and veins as compared with levels in maternal circulation of normoglycemic mothers (23.8 ± 16.9 ng/mL and 22 ± 13.9 ng/mL for umbilical artery and vein, respectively, vs. 15.2 ± 7.6 ng/mL in normoglycemic women, *P* < 0.05 for both) (Figure 1C). Neonatal FABP4 levels collected within the first 6 hours of birth demonstrated a further rise, reaching a marked approximately 3-fold increase compared with fetal levels (mean value of 65.6. ± 32 ng/mL, *P* < 0.001, Figure 1D). This rapid increase in FABP4 levels in neonates immediately after birth is in accordance with the time frame of activation in hepatic glucose production previously reported in newborns (7, 12). A nonsignificant trend toward reduced FABP4 levels was observed in neonates who were large for gestational age (LGA) (Figure 1E). Of note, FABP4 levels in neonates did not correlate with birth weight (Figure 1F).

### Neonatal FABP4 levels inversely correlate with blood glucose.

**Figure 2:**
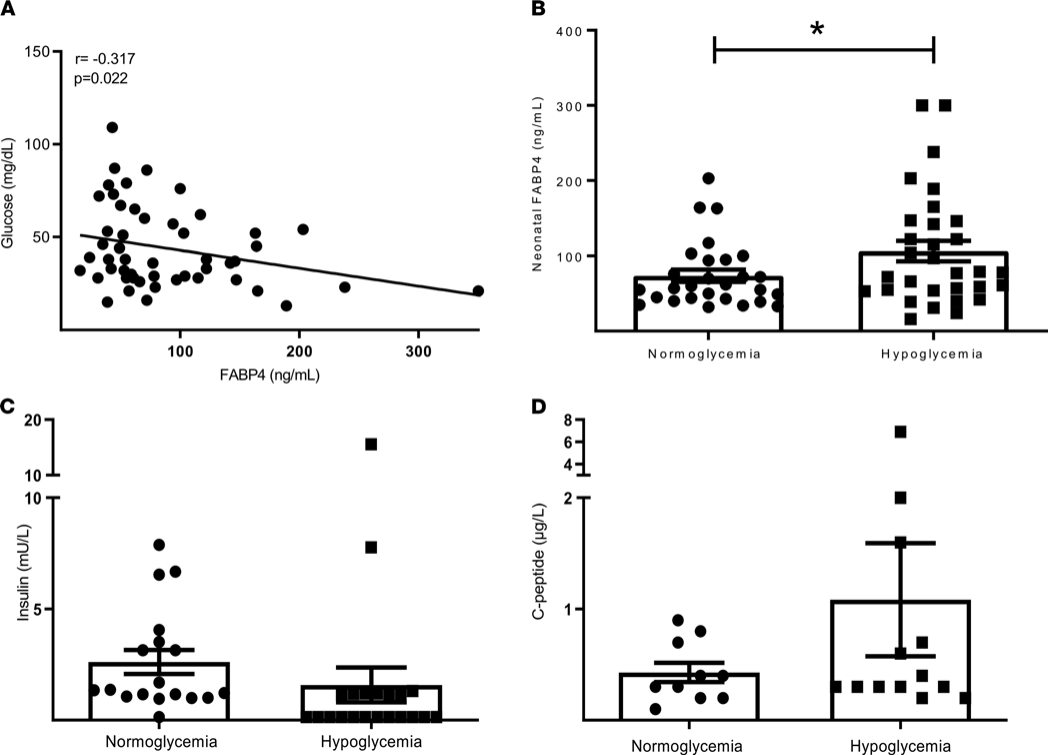
Neonatal FABP4 levels inversely correlated with blood glucose and are elevated among hypoglycemic neonates. Serum samples collected from 21 normoglycemic and 29 hypoglycemic neonates (as detailed in Table 2) within the first few hours of life. (**A**) FABP4 circulating levels in neonates were correlated with blood glucose levels. Comparison of serum FABP4 between 31 normoglycemic and 28 hypoglycemic neonates is presented in (**B**). (**C** and **D**) C-peptide circulating levels in neonates were compared and analyzed between normoglycemic (*n* = 10) and hypoglycemic (*n* = 13) neonates (**D**) and insulin circulating levels in neonates were compared and analyzed between normoglycemic (*n* = 19) and hypoglycemic neonates (*n* = 21) (**C**). Statistical analysis was performed by nonparametric correlations using Spearman’s correlation test (**A**) and by Student’s *t* test (**B–D**). Data are shown as the mean ± SEM. *P < 0.05.

Because the level of circulating FABP4 has been shown to be regulated by fasting and lipolysis-related signals and, thus, to promote hepatic glucose production (14), we assessed the association of neonatal serum FABP4 with blood glucose, demonstrating a direct inverse correlation. As depicted in Figure 2A, neonatal FABP4 levels inversely correlated with blood glucose (Spearman’s *r* = –0.317, *P* = 0.022), with significantly elevated levels recorded among neonates who developed hypoglycemia (blood glucose, <40 mg/dL), reaching a mean value of 106.2 ± 13.6 ng/mL, compared with 73.6 ± 8.2 ng/mL in normoglycemic neonates (*P* = 0.05, Figure 2B). Insulin (Figure 2C) and C-peptide (Figure 2D) levels were not significantly different between normoglycemic neonates and neonates who developed hypoglycemia. The normal, unsuppressed levels of insulin, inappropriately observed in hypoglycemic neonates, further support the role of altered regulation of insulin secretion as an important contributor to postnatal hypoglycemia (23).

### FABP4 affects glucose homeostasis in mouse neonates.

**Figure 3:**
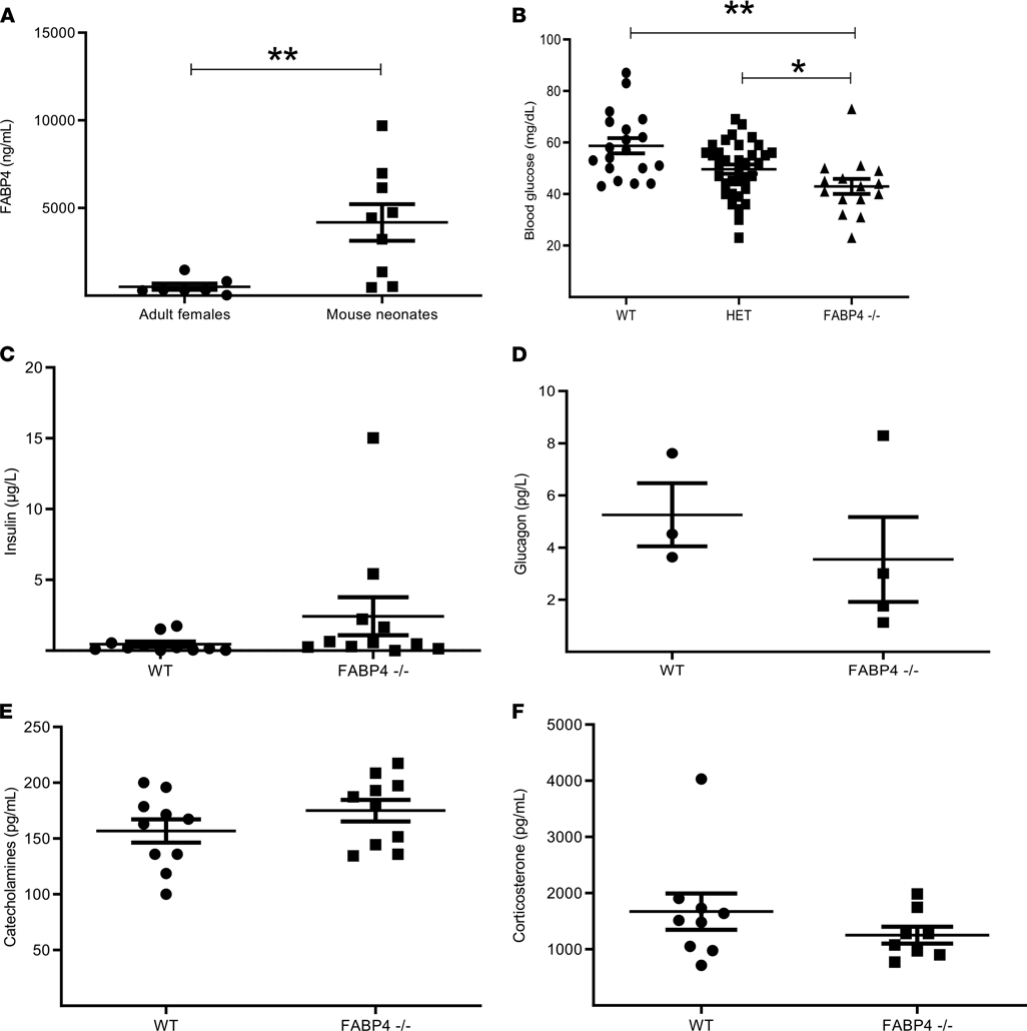
Levels of FABP4 and its effect on blood glucose, insulin, and hormonal network in mouse neonates. Plasma samples were collected from 9 WT mouse neonates within 12 hours from birth and 7 adult WT female mice. (**A**) FABP4 concentrations were determined using an ELISA assay and were compared between neonates and adult mice. (**B**) Mice heterozygous for Fabp4-null mutation were crossbred, and the offspring’s fasting blood glucose levels were tested 12 hours following birth. Levels of Fabp4^WT^ (*n* = 19), heterozygous (*n* = 35), and Fabp4^–/–^ (*n* = 15) mice were compared. (**C**) Insulin (of 11 Fabp4^WT^ and 11 Fabp4^–/–^ mouse neonates), (**D**) glucagon (of 3 Fabp4^WT^ and 4 Fabp4^–/–^ mouse neonates), (**E**) catecholamines (of 10 Fabp4^WT^ and 10 Fabp4^–/–^ mouse neonates), and (**F**) corticosterone (of 9 Fabp4^WT^ and 8 Fabp4^–/–^ mouse neonates) plasma levels were compared between Fabp4^WT^ and Fabp4^–/–^ mouse neonates within 12 hours following birth. Statistical analysis was performed by Student’s *t* test (**A** and **C–F**) and by 1-way ANOVA test (**B**). Data are shown as the mean ± SEM. *P < 0.05, **P < 0.01. FABP4^WT^; WT, HET; heterozygous.

To further study FABP4 dynamics and role in neonatal glucose metabolism, we tested its plasma levels in mouse neonates within 12 hours from delivery. The results demonstrated a significant increase of about 8-fold in neonatal FABP4 plasma levels in comparison with those of adult females (mean value of 509.3 ± 480.3 ng/mL vs. 4178. ± 3136 ng/mL, *P* < 0.001, Figure 3A), which resembles the results in humans (Figure 1D). To further demonstrate a direct role for FABP4 in regulating glucose hemostasis, we assessed neonatal glucose levels in *Fabp4*^–/–^ mice. Following crossbreeding of mice heterozygous for *Fabp4*, we tested blood glucose of WT (*Fabp4^WT^*), *Fabp*4^–/+^ heterozygote, and *Fabp4*^–/–^ littermates. Blood glucose of approximately 12-hour-old mice was measured, with a mean level of 58.7 ± 12.8 mg/dL recorded among WT mice, 49.6 ± 10.6 mg/dL in *Fabp4*^–/+^ mice, and 42.9 ± 11.3 mg/dL in *Fabp4*^–/–^ mice (*P* < 0.01 between WT and *Fabp4*^–/–^ mice, *P* < 0.05 between WT and *Fabp4*^–/+^ mice, Figure 3B). Of note, similar to observations in humans (Figure 2A), FABP4 levels inversely correlated with blood glucose in *Fabp4^WT^* neonates (Pearson’s *r* = –0.460, *P* = 0.03). Because the induction of gluconeogenesis and hepatic glucose production following delivery involves the coordinated activation of an endocrine hormonal network, including increased plasma levels of catecholamines, glucagon, and glucocorticoids (9, 10), we tested plasma concentrations of insulin (Figure 3C), glucagon (Figure 3D), catecholamines (Figure 3E) and corticosterone (Figure 3F) in *Fabp4^WT^* and *Fabp4*^–/–^ neonates within 12 hours after delivery, with no significant differences observed between *Fabp4^WT^* and *Fabp4*^–/–^ neonates in any of these hormones.

### FABP4 effect on gluconeogenic genes.

**Figure 4:**
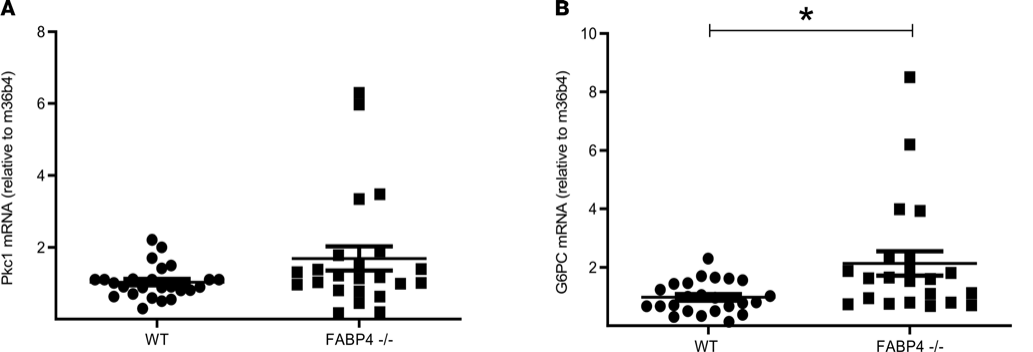
Hepatic glucose production in *Fabp4^WT^* and *Fabp4^–/–^* mouse neonates. Expression levels of key gluconeogenic genes in livers of mouse neonates: phosphoenolpyruvate carboxykinase 1 (Pck1) (*n* = 25 for *Fabp4^WT^* and 23 *Fabp4^–/–^*) (**A**) and glucose-6-phosphatase (G6pc) (*n* = 23 for *Fabp4^WT^* and 22 *Fabp4^–/–^*) (**B**). Statistical analysis was performed by Student’s *t* test. Data are presented as mean ± SEM. *P < 0.05.

Because FABP4 has been shown to mediate hepatic glucose production in mature mice, and was suggested to regulate the expression of key gluconeogenic genes (14), we studied whether the decreased blood glucose in *Fabp4*^–/–^ neonates could be explained by reduced liver mRNA expression of phosphoenolpyruvate carboxykinase 1 (Pck1) (Figure 4A) and glucose-6-phosphatase (G6pc) (Figure 4B). Of note, the expression of neither of these genes was reduced, with even a significant increase in G6pc mRNA levels observed in livers of *Fabp4*^–/–^ mice, which may represent a compensatory adaptation to hypoglycemia in *Fabp4*^–/–^ mice.

### The effect of FABP4 on the neonatal metabolic transcriptional network.

**Figure 5:**
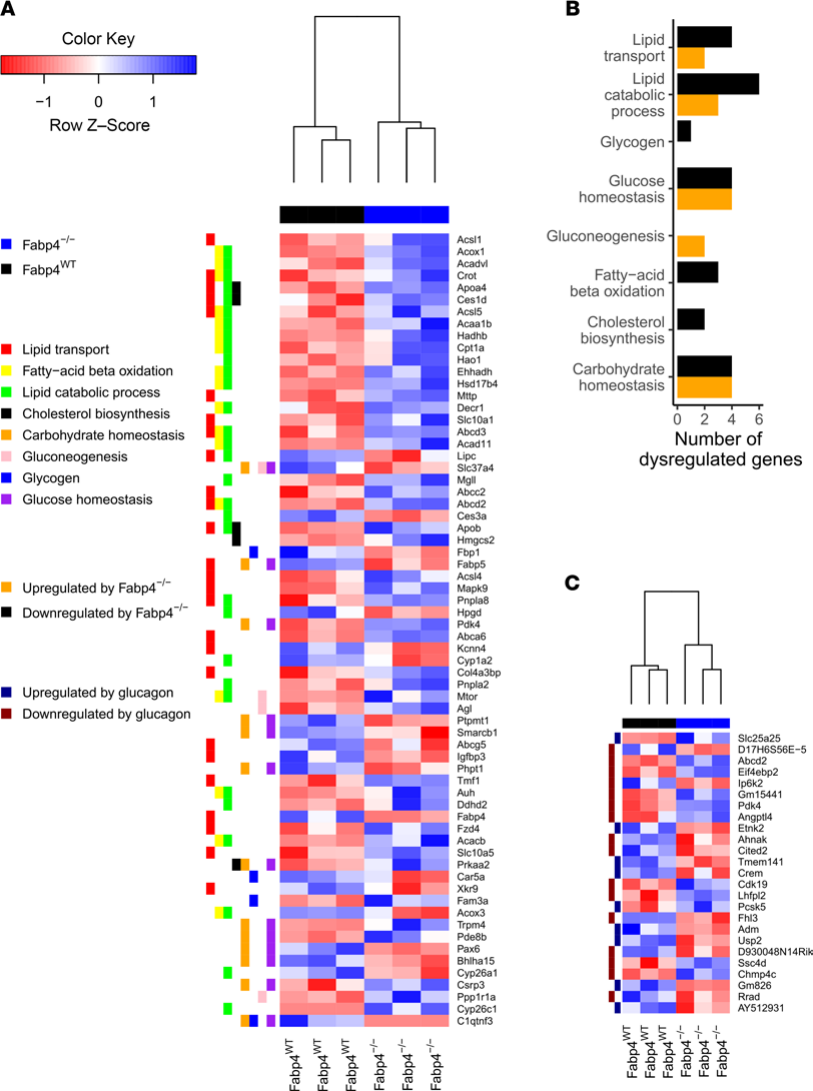
Differential hepatic gene expression analysis of FABP4-deficient mice. Differential hepatic gene expression analysis showing the effect of the FABP4-deficient state on various lipid- and carbohydrates-related pathways and the over/underexpression of genes in each related pathway. (**A**) Heatmap. (**B**) Bar chart. (**C**) The effect of FABP4 on genes that are known to be regulated in glucagon-treated hepatocytes. *n* = 3 in each study group.

In an attempt to better understand the potential contribution of FABP4 to the regulation of postnatal metabolic homeostasis, we subjected livers from 48-hour-old *Fabp4*^–/–^ and *Fabp4^WT^* mice, following 4 hours of starvation, to RNA sequencing. We performed targeted analysis of the key pathways involved. As demonstrated in Figure 5A , the absence of *Fabp4* resulted in altered regulation not only of lipid homeostasis pathways, as one would expect given the well-characterized role of FABP4 as a lipid chaperon, but also of key pathways regulating hepatic glucose production. Of note, both gluconeogenesis and glycogen metabolism seemed to be affected by *Fabp4* deficiency and could account, at least partly, for the hypoglycemic phenotype. Given the key regulatory role of glucagon in many of these pathways, and our previous results demonstrating impaired glucagon bioactivity in the absence of FABP4 (24), we next assessed directly the differential expression of glucagon-regulated genes in livers of *Fabp4*^–/–^ and *Fabp4^WT^* mice. Indeed, analyzing genes that were directly regulated by glucagon revealed altered expression in *Fabp4*^–/–^ mice (Figure 5, B and C). Furthermore, 3 genes were downregulated by glucagon and upregulated by *Fabp4*^–/–^. These included Gm15441, encoding a lncRNA, which is involved in regulating fatty acid oxidation in hepatocytes (25), and glucose homeostasis during fasting (26); pyruvate dehydrogenase kinase 4 (Pdk4), with a known key role in fatty acid oxidation and in glucagon regulation of hepatic glucose production (27); and Abcd2, a regulator of fatty acid homeostasis (28). These findings further support the potential link between Fabp4 and glucagon and their coassociation with key metabolic processes.

### FABP4 effect on liver glycogen content.

**Figure 6:**
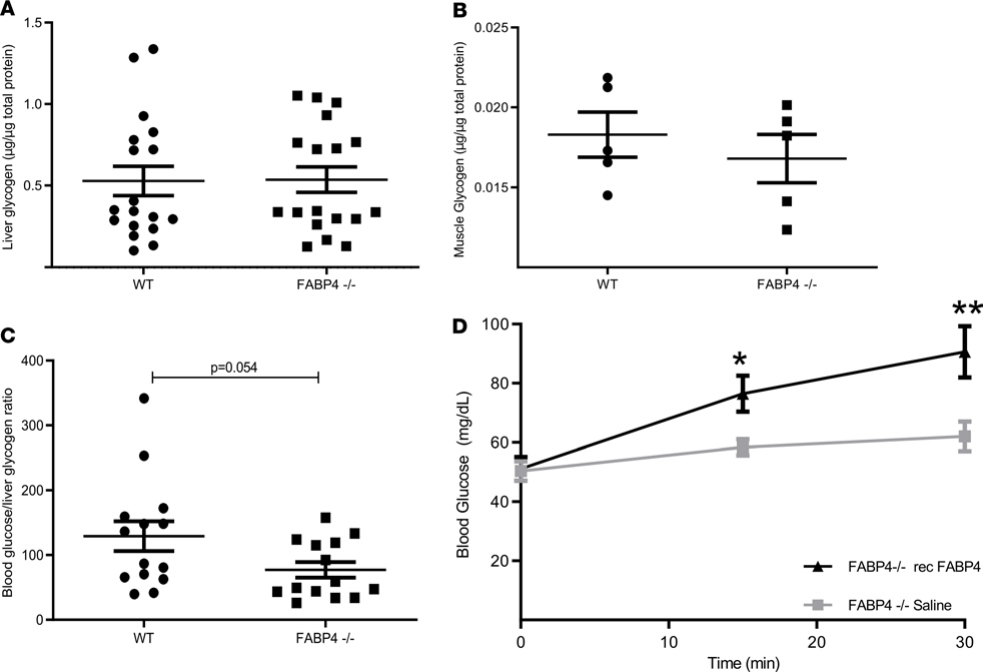
Glycogen content of *Fabp4^WT^* and *Fabp4^–/–^* mouse neonates and recombinant FABP4 effect on blood glucose. *Fabp4^WT^* and *Fabp4^–/–^* liver and muscle (left thigh) tissues were harvested and snap frozen at liquid nitrogen. (**A** and **B**) Glycogen content was determined in Fabp4^WT^ (*n* = 19) and *Fabp4*^–/–^ (*n* = 18) mouse neonatal liver (**A**) and *Fabp4^WT^* (*n* = 5) and *Fabp4*^–/–^ (*n* = 5) mouse neonatal muscle (**B**) tissues using the Glycogen Assay Kit. (**C**) The ratio between blood glucose and liver glycogen content in each subject was compared between *Fabp4^WT^* (*n* = 14) and *Fabp4^–/–^* (*n* = 14) neonates. Recombinant FABP4 or saline was injected in *Fabp4^–/–^* mice (*n* = 7 for FABP4 and 8 for saline injected) within 12 hours from delivery. (**D**) Blood glucose was measured from mouse tail vein at 0, 15, and 30 minutes following injections. Statistical analysis was performed by Student’s *t* test. Data are presented as mean ± SEM. *P < 0.05, **P < 0.01.

As FABP4 deficiency in mouse neonatal liver tissues resulted in alterations of glucagon bioactivity, including pathways involved in glycogen metabolism and glucagon receptor–regulated genes as explained above, we tested glycogen content in neonates of *Fabp4^WT^* and *Fabp4*^–/–^ mice. The results showed that, despite a marked hypoglycemia in *Fabp4*^–/–^ mice, liver glycogen content was comparable to that of normoglycemic *Fabp4^WT^* controls (Figure 6A). Similar glycogen content was also observed in skeletal muscle of *Fabp4^WT^* and *Fabp4*^–/–^ mice (Figure 6B). Thus, the inappropriately intact liver glycogen stores in the setting of substantial hypoglycemia argues in favors of impaired glucagon-induced glycogenolysis in *Fabp4*^–/–^ mice. Indeed, a lower ratio of blood glucose to liver glycogen was observed in *Fabp4*^–/–^ compared to *Fabp4^WT^* mice (Figure 6C, *P* = 0.054).

### Introduction of FABP4 to Fabp4^–/–^ mouse neonates restores normoglycemia.

In order to assess the direct contribution of FABP4 in maintaining neonatal normoglycemia, we injected recFABP4 or saline in *Fabp4*^–/–^ mouse neonates within 12 hours from delivery. Of note, no significant differences in body weight were observed between *Fabp4^WT^* (1.34 ± 0.12 g) and *Fabp4*^–/–^ (1.38 ± 0.13 g) mice (*P* = 0.39). As demonstrated in Figure 6D, while subcutaneous saline injection did not significantly change blood glucose levels in *Fabp4*^–/–^ neonates, injection of recFABP4 resulted in a rapid and significant rise in blood glucose levels and restored normoglycemia within 15 minutes, reaching an approximately 2-fold increase in 30 minutes following injection (Figure 6D).

## Discussion

FABP4 is an intracellular lipid chaperon, expressed mainly in adipocytes with a key role in intracellular lipid metabolism and signaling (13). Circulating FABP4 has been demonstrated to promote hyperglycemia and to stimulate hepatic glucose production by regulating expression of key gluconeogenic enzymes (14). In the current study, we tested the role of FABP4 in neonatal glucose homeostasis by studying the dynamics in its circulating levels in maternal, fetal, and neonatal states. Additionally, we validated its mechanistic role in maintaining glucose homeostasis in a genetic mouse model with either haploinsufficiency or complete deletion of *Fabp4*.

Following delivery, there is a rapid shift in the neonate metabolism from high-carbohydrate and low-fat energy supply to high-fat– and low-carbohydrate–based nutrition. During normal pregnancy, given a constant transplacental glucose supply, fetal hepatic glucose production is essentially absent (6). The abrupt cessation of placental energy supply after delivery obligates a rapid and efficient induction of hepatic glucose production in order to maintain euglycemia. This is accomplished by activation of an endocrine hormonal network, which includes a rise in plasma catecholamines (~3- to 10-folds), glucagon (~3- to 5-folds), and cortisol (9, 10). In addition, an increase in liver expression of the glucagon receptor has also been demonstrated to occur shortly following delivery (9, 10). In parallel, insulin levels decrease, which leads to a low insulin/glucagon ratio, further triggering the induction of hepatic glycogenolysis and gluconeogenesis activation (9, 10). Yet, the mechanisms of this punctual regulated process are not fully understood (12).

FABP4 levels in the fetal circulation (both in the umbilical artery and vein) were significantly higher than those observed in normoglycemic women; therefore, fetal circulation likely represents a source for FABP4. An additional spike in FABP4 levels was observed in both human and mouse neonates within few hours after birth. This rise in FABP4, which correlates with the drop in blood glucose observed within the first day of life, may indicate an additional hormonal signal in the complex insulin counterregulatory endocrine network, aiming at preventing postnatal hypoglycemia (9, 10). Indeed, the highest levels of FABP4 were recorded in neonate who developed hypoglycemia.

Another observation in the current study is a nonsignificant decrease in FABP4 levels in neonates who were LGA, which is somewhat in contrast to a positive correlation between FABP4 and body mass observed in children and adults. This observation may reflect fetal hyperinsulinism, a key driver for LGA and macrosomia (29, 30), which is also known to suppress FABP4 levels. The suppression of adequate rise in FABP4 among LGA neonates may further exacerbate the risk for hypoglycemia associated with LGA (31). While a negative correlation between birth weight and umbilical cord FABP4 levels was similarly reported in a cohort of neonates born at term (32), an opposite trend was observed in preterm neonates demonstrating somewhat lower FABP4 levels in infants who were small for gestational age (SGA) (33). While still elevated, the relative suppression of the rise in FABP4 in preterm SGA infants, largely reflecting in utero pathologies, could potentially be explained by much lower fat mass and decreased secretory capacity of the adipose tissue. It is also important to mention that, in addition to adipocytes, FABP4 has been demonstrated to be expressed and secreted by macrophages (34) and vascular endothelial cells (35). The expression and secretion of FABP4 in macrophages are associated with their immune activation (36), which is less likely to occur during acute hypoglycemia. In addition, the predominant contributors of circulating FABP4 are likely to be adipocytes rather than macrophages or endothelial cells (14, 37).

The rise in postnatal FABP4 levels, the observed peak in glucagon and expression of the liver glucagon receptor, and the altered liver expression of glucagon-regulated genes in the absence of FABP4 are intriguing. We have recently demonstrated a potential interaction between the biological activities of both FABP4 and glucagon (24). In that study, the hyperglycemic effect of propionate required both glucagon and FABP4. In fact, glycogenolysis and subsequent hyperglycemia could be almost completely prevented when Fabp4 was either genetically deleted or pharmacologically neutralized, despite an intact increase in glucagon level (24). Thus, it is possible that FABP4 may augment glucagon-induced glycogen breakdown and glucagon-regulated gene expression, by serving as a coactivator of glucagon actions and as a bona fide insulin counterregulatory endocrine signal (24). Indeed, despite a marked hypoglycemia observed in *Fabp4*^–/–^ neonates, liver glycogen stores remained largely intact. While this could not be explained by decreased levels of glucagon, catecholamines, and corticosterone, impaired expression of glucagon-regulated genes suggested an altered glucagon activity in the absence of *Fabp4*, which may also result in defective glycogenolysis. The immediate correction of hypoglycemia by injection of recFABP4 further supports a necessary hormonal role for FABP4 in maintaining normoglycemia by correcting the impaired glucagon-mediated glycogenolysis as we previously suggested (24). Thus, the increase in circulating FABP4 levels after birth may represent a yet-unrecognized component in the orchestrated hormonal and metabolic adaptive response to the abrupt metabolic switch of birth. In this adaptive network, which includes a vast increase in circulating glucagon, catecholamines, and cortisol (1), FABP4 may act as an adipocyte-derived coactivator, leading both to increase in the biological activities of glucagon as well as ensuring adequate delivery of free fatty acids as an energy source to the glucose producing liver.

While elevated FABP4 has been well demonstrated in obesity, the metabolic syndrome, and type 2 diabetes and likely contributes to the well-documented insulin resistance in these pathological conditions, to the best of our knowledge this is the first report demonstrating an important physiological role of this adipokine.

Taken altogether, the significant increase in fetal FABP4 levels immediately before delivery, which continue to increase during the first few hours of life, suggests a potential role for this adipokine in the complex postnatal regulation of glucose metabolism and the rapid induction of neonatal hepatic glucose production. The robust increase in circulating FABP4 during hypoglycemia and its ability to restore normoglycemia may indicate a role for FABP4 as an additional insulin counterregulatory hormone.

## Methods

### Patients and samples collection.

Serum samples were collected from 22 normoglycemic pregnant women and 18 women with GDM (10 women with GDM controlled by diet and exercise [GDMA1] and 8 women with GDMA2). A “2-step” method (according to the International Association of Diabetes and Pregnancy Study Groups criteria) was employed to diagnose GDM. Pregnant women were diagnosed as having GDM if they failed the glucose challenge test (GCT), with a cut-point of 140 mg/dL (7.8 mmol/L), in addition to having 1 or more abnormal values from the 3-hour 100 g oral glucose tolerance test (OGTT) between 24 and 28 weeks of gestation. The cut-points of the OGTT were 95 mg/dL (5.3 mmol/L) at fasting, 180 mg/dL (10 mmol/L) at 1 hour, 155 mg/dL (8.6 mmol/L) at 2 hours, and 140 mg/dL (7.8 mmol/L) at 3 hours after glucose load. Pregnant women with normal GCT (and/or OGTT, if performed) were classified as normoglycemic (control group).

All samples were collected at term before delivery and from the umbilical artery and vein immediately after birth. Determination of blood glucose and FABP4 level in newborns within the first few hours of life was made possible by using samples collected for national screening tests performed in all newborns. In addition, we also collected serum samples from 26 neonates who developed hypoglycemia (capillary blood glucose <40 mg/dL) within the first few hours of life, for whom we did not have maternal samples. The baseline characteristics of the maternal GDMA1 and GDMA2 groups as well as the control group are summarized in Table 1. Characteristics of the hypoglycemic neonates and normoglycemic controls are presented in Table 2.

### Anthropometric measurements.

Anthropometric data comprised prepregnancy weight and height. Standing height was measured barefoot to the nearest 1 cm, and body weight was obtained to the nearest 1 kg. BMI was calculated by weight (Kg)/squared height (m)^2^.

### FABP4, insulin, C-peptide, and glucose measurements in human neonates.

FABP4 (Human FABP4 Quantikine ELISA Kit DFBP40, R&D Systems) and insulin (Mercodia Insulin ELISA, Mercodia) concentrations were determined in serum samples using ELISA according to the manufacturer’s instructions. Human C-peptide concentrations in plasma were determined by ELISA (IMMULITE 2000 C-Peptide) using an IMMULITE 2000 Immunoassay System (Siemens Medical Solutions Diagnostics). Glucose levels were determined using Cobas b 211 (Roche Diagnostics).

### Animal housing and treatments.

C57Bl/6 mice with homozygous null mutations in Fabp4 were provided to us by G. Hotamisligil, at the Sabri Ulker Center, Harvard T.H. Chan School of Public Health, Boston, Massachusetts, USA. Mice were bred and housed at Sheba medical center facility on a 12-hour-light/dark cycle. Up to 12 hours following parturition, between 7 and 10 am, littermates were separated from lactating females for 2 hours. Following decapitation, blood was collected and kept on ice. Plasma was collected from decapitation bleeding followed by rapid harvesting of liver and muscle tissues. Tissues were snap frozen at liquid nitrogen.

### Mouse FABP4 and counterregulatory hormone circulating concentrations.

Mouse FABP4 (Mouse FABP4/A-FABP ELISA, RayBiotech), insulin (Insulin ELISA Mouse UltraSensitive, Mercodia), glucagon (Glucagon ELISA Human/Rat/Mouse, Mercodia), catecholamines (QuickDetect Catecholamine, Biovision), and corticosterone (Corticosterone ELISA Kit, Arbor Assays) concentrations were determined in mouse neonatal plasma samples using ELISAs according to the manufacturer’s instructions.

### Mouse neonatal liver and muscle glycogen content.

Liver and muscle (whole body) tissues were snap-frozen in liquid nitrogen until sample processing. Upon sample processing, liver and muscle (left thigh of each mouse neonate) tissues were boiled immediately in 120 μl DDW and subjected to Bradford assay to determine protein concentrations. Glycogen content was determined in 3 μg total extract using Glycogen Assay Kit (Abcam Plc.), according to manufacturer’s instructions.

### Blood glucose levels of mouse neonates.

Blood glucose levels of mouse neonates were determined from tail vein using FreeStyle strips (Abbott Laboratories).

### RNA preparation and gene expression.

Total RNA was isolated from mouse neonatal liver using TRIzol Reagent (Ambion, Life Technologies, Thermo Fisher Technologies) according to the manufacturer’s protocol. First-strand cDNA was synthesized from 0.5 to 1 μg total RNA using qScript cDNA synthesis kit (Quanta bio) according to the manufacturer’s instructions. Gene expression was determined by quantitative real-time PCR, and the results were analyzed using StepOnePlus Real-Time PCR Systems program (Thermo Fisher Scientific).

Primers used in the study were purcahsed from Integrated DNA Technologies (IDT Synthesis) and are detailed in Table 3.

### RNA sequencing.

RNA from liver tissue samples of *Fabp4^WT^* and *Fabp4*^–/–^ mouse neonates (3 samples each) was extracted as described above. Samples processed for RNA sequencing had RNA integrity score of ≥6 for all samples. Paired-end RNA sequencing was performed on an Illumina platform and processed by trimming (trimmomatic; ref. 38), alignment (STAR, ref. 39; using mm10 as the reference genome) and expression quantification (HTSeq-count; refs. 40, 41) and differential expression analysis by DESeq2 (41). RNA-sequencing result validity was assessed by comparing qPCR results of G6pc that demonstrated similar differential expression (*P* = 0.07). To assess the effect of transcriptome changes of *Fabp4^–/–^* liver tissue, targeted analysis of the key pathways involved was performed, based on the Kyoto Encyclopedia for Genes and Genomes (KEGG) database. Figure 5, A and B, are based solely on these data.

The effect of the FABP4-deficient state on expression of glucagon-regulated genes was assessed in a separate analysis. Primary mouse hepatocytes were isolated from male 8-week-old C57Bl/6 mice as previously described (42). Cells were cultured in low-glucose DMEM (catalog 11885 Gibco, Thermo Fisher Scientific). One day after isolation, hepatocytes were treated with 10 nM glucagon (catalog G2044, MilliporeSigma) for 2 hours, RNA was extracted (catalog 740955.25 NucleoSpin, Macherey-Nagel), and RNA-sequencing analysis was performed. Glucagon-regulated genes were determined with these cutoffs: fold change ≥ 1.5, with adjusted *P* ≤ 0.05 defined as upregulated (*n* = 359) or downregulated genes (*n* = 325). Data analysis and figures were produced on R studio version 1.4.1106 (PBC). Figure 5C is based on cross analysis of the *Fabp4*^–/–^ versus WT differential gene expression analysis and the glucagon stimulation study results. The raw data files were uploaded to Dryad public data repository (doi:10.5061/dryad.sbcc2fr6x).

### recFABP4 injections in mouse neonates.

Mouse neonates were studied within 12 hours from delivery, and blood glucose levels were determined from the tail vein using FreeStyle glucose strips (Abbott Laboratories). Fifty μg/kg recFABP4 (AG-40A-0035-C050, AdipoGene, Life Sciences) (24) or saline was injected subcutaneously, and glucose was measured from tail veins at 15 and 30 minutes following injections.

### Statistics.

Statistical analyses were performed on GraphPad Prism9 (Graphpad Software). Continuous variables are presented as mean ± standard error of mean (SEM). Student’s 2-tailed *t* test was used to compare serum FABP4 between hypoglycemic and normoglycemic neonates (Figure 2B), fasting glucose levels and Apgar values (Table 2); C-peptide and insulin circulating levels in neonates (Figure 2, C and D); FABP4 serum levels, insulin, glucagon, catecholamines and corticosterone in mice (Figure 3, A, and C–F); expression of genes in livers of mice neonates (Figure 4); glycogen content and glucose levels following recFABP4 injection (Figure 6). One-way ANOVA was used to compare FABP4 levels in the maternal circulation and in the umbilical vein and artery (Figure 1C), as well as to compare FABP4 levels between matched maternal (Figure 1D), umbilical and neonatal samples and between neonates who were small, appropriate or large for gestational age (SGA, AGA and LGA, respectively) (Figure 1E), FABP4 serum levels between normoglycemic, GDMA1, GDMA2 pregnant women (Figure 1B); Table 1; fasting blood glucose in different FABP4 genotypes (Figure 3B). To compare the rates of SGA, AGA, and LGA between normoglycemic and hypoglycemic neonates (Table 2), we used the χ^2^ test. Nonparametric correlations between variables were performed using Spearman’s correlation test. Two-tailed *P* values of less than 0.05 were used for defining statistical significance

### Study approval.

The Institutional Review Board of Sheba Medical Center in Israel approved the study protocol, and all participants provided written informed consent. All animal experimental protocols were approved by the Institutional Animal Care and Use Committee of Sheba Medical Center (1310/21/ANIM).

## Author contributions

TS, IMP, KO, and Amir Tirosh designed the study. RKL and KO obtained informed consent and collected data. IR, RKL, MR, RH, and EB performed laboratory assays and data analyses. MR, RL, and SR performed animal studies. RKL, IR, IMP, Amit Tirosh, and Amir Tirosh interpreted data. Amit Tirosh and IG performed bioinformatics analyses. IR, IMP, and Amir Tirosh drafted the manuscript. All authors reviewed, edited and approved the final version of the manuscript. IMP, RKL, and Amir Tirosh have full access to all the data in the study and take responsibility for the integrity of the data and the accuracy of the data analysis.

## Figures and Tables

**Table 1:**
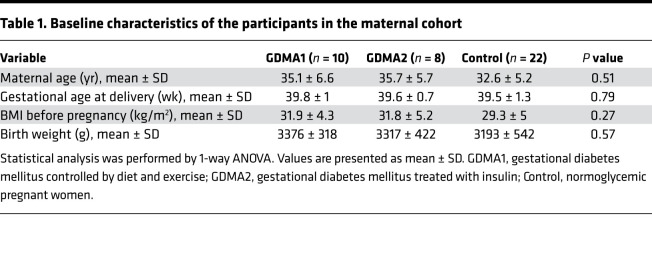
Baseline characteristics of the participants in the maternal cohort

**Table 2:**
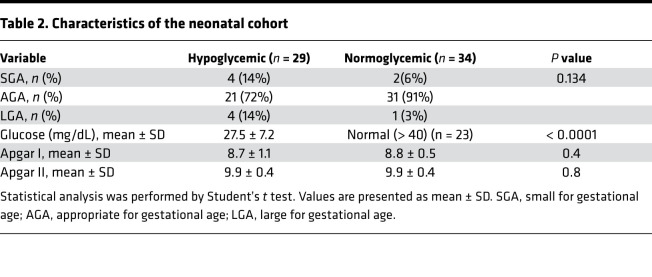
Characteristics of the neonatal cohort

**Table 3:**
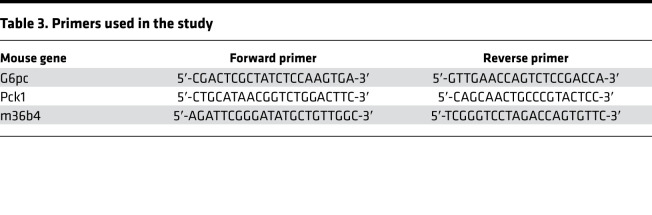
Primers used in the study

## Version 1 10/22/2021

Electronic publication
